# THISTLE: trial of hands-on Interprofessional simulation training for local emergencies: a research protocol for a stepped-wedge clustered randomised controlled trial

**DOI:** 10.1186/s12884-017-1455-9

**Published:** 2017-09-07

**Authors:** Erik Lenguerrand, Catherine Winter, Karen Innes, Graeme MacLennan, Dimitrios Siassakos, Pauline Lynch, Alan Cameron, Joanna Crofts, Alison McDonald, Kirsty McCormack, Mark Forrest, John Norrie, Siladitya Bhattacharya, Tim Draycott, On behalf of the Thistle group

**Affiliations:** 1 School of Clinical Sciences, University of Bristol, Level 1 Learning and Research, Southmead Hospital, Bristol, BS10 5NB UK; 2 PROMPT Maternity Foundation: registered charity in England & Wales, No:1140557, London, UK; 3 Centre for Healthcare Randomised Trials (CHaRT), University of Aberdeen, Aberdeen, AB25 2ZD UK; 4 North Bristol Trust, Department of Women’s Health, The Chilterns, Southmead Hospital, Bristol, BS10 5NB UK; 5 NHS Tayside, Maternity Unit, Ninewells Hospital and Medical School, Dundee, DD2 1UB UK; 6 Ian Donald Fetal Medicine Centre, Queen Elizabeth University Hospital, G51 4TF, Glasgow, UK; 7 Edinburgh Clinical Trials Unit, University of Edinburgh, Western General Hospital, Edinburgh, EH4 2XU UK; 8 Institute of Applied Health Sciences, University of Aberdeen, Aberdeen, AB25 2ZD UK; 9 School of Social and Community Medicine, University of Bristol, Department of Women’s Health, The Chilterns, Southmead Hospital, Bristol, BS10 5NB UK

**Keywords:** Intrapartum emergencies, Training course, Apgar score, Stepped-wedge trial, Obstetrics

## Abstract

**Background:**

Many adverse pregnancy outcomes in the UK could be prevented with better intrapartum care. Training for intrapartum emergencies has been widely recommended but there are conflicting data about their effectiveness. Observational studies have shown sustained local improvements in perinatal outcomes associated with the use of the PRactical Obstetric Multi-Professional Training – (PROMPT) training package. However this effect needs to be investigated in the context of randomised study design in settings other than enthusiastic early adopter single-centres. The main aim of this study is to determine the effectiveness of PROMPT to reduce the rate of term infants born with low APGAR scores.

**Methods:**

THISTLE (Trial of Hands-on Interprofessional Simulation Training for Local Emergencies) is a multi-centre stepped-wedge clustered randomised controlled superiority trial conducted across 12 large Maternity Units in Scotland. On the basis of prior observational findings all Units have been offered the intervention and have been randomly allocated in groups of four Units, to one of three intervention time periods, each six months apart.

Teams of four multi-professional clinicians from each participating Unit attended a two-day PROMPT Train the Trainers (T3) programme prior to the start of their allocated intervention step. Following the T3 training, the teams commenced the implementation of local intrapartum emergency training in their own Units by the start of their allocated intervention period. Blinding has not been possible due to the nature of the intervention. The aim of the study is to follow up each Unit for at least 12-months after they have commenced their local courses.

The primary outcome for the study is the proportion of Apgar scores <7 at 5 min for term vaginal or emergency caesarean section births (≥37 weeks) occurring in each of the study Units. These data will be extracted from the Information Services Division Scottish Morbidity Record 02, a national routine data collection on pregnancy and births. Mixed or marginal logistic regression will be employed for the main analysis.

**Discussion:**

THISTLE is the first stepped wedge cluster randomised trial to evaluate the effectiveness of an intrapartum emergencies training programme. The results will inform training, trainers and policy going forward.

**Trial registration:**

ISRCTN11640515 (registered on 09/09/2013).

## Background

Safety in labour is a priority for women, their families, staff and the National Health Service (NHS), but UK maternity care is not as safe as it could, and should be. Perinatal outcomes are substantially worse than those in countries with similar Gross Domestic Product [1], and rates of maternal death, stillbirth, neonatal death, neonatal injury and cerebral palsy are higher than those reported from many developed countries. It is believed that over 50% of adverse pregnancy outcomes in the UK could be prevented with better intrapartum care [1–3] thus benefiting families as well as the NHS which spends more than half of its total litigation costs on maternity services, amounting to £3.1bn between 2000 and 2010 [4].

Training for intrapartum emergencies has been recommended consistently since 1924, and almost annually since 1999 [1–3, 5, 6]. While some training reports have either failed to demonstrate any change or shown deterioration [7–9], others from single maternity Units have suggested post–training improvements in individual outcomes [7, 8, 10–13].

Observational studies of a local, multi-professional intrapartum emergencies training course for local maternity staff – PRactical Obstetric Multi-Professional Training – (PROMPT, Bristol, UK) have all shown positive effects. Its implementation was associated with improved compliance with clinical standards [10, 12] and a reduction in clinical error [11]. There is also evidence of improvements in perinatal outcomes including a 70% reduction in brachial plexus injuries (paralysed arm) and a 50% reduction in neonatal hypoxic-ischaemic encephalopathy (predictor of cerebral palsy) [10, 11]. The prevalence rate of 5-min Apgar (Appearance, Pulse, Grimace, Activity, Respiration) scores <7 has reduced by 48% from 0.87% to 0.45% following training and the retention of clinical skills in maternity staff at 12 months were excellent [14]. These improvements in outcomes after training have been sustained since 2004 [15]. More importantly, similar advances were also observed in three other settings, Melbourne Australia [16], Kansas USA [17], and Zimbabwe [18].

Clinical benefits demonstrated in small studies can prove to be less impressive when applied at scale [19]. While these early data for PROMPT training are extremely encouraging, it is important to determine whether these improvements can be sustained outside the host Unit and enthusiastic early adopters, ideally across a health system.

### Rationale for the trial

There is currently no definitive evidence to recommend a specific training programme for intrapartum emergencies to improve safety in labour. PROMPT is the only training package for which several observational studies, mostly single-centre but conducted in different settings have shown consistent improvement in key perinatal outcomes. Stronger evidence from a more robust multicentre study is now required to ascertain its effectiveness, especially at scale.

Scotland has over 50,000 births annually [20] and provides an ideal setting to study the effect of implementation of training at scale, as only 3 of the 15 Scottish Maternity Units (with births per year >900, 2012 data) have previously undertaken PROMPT training. Local maternity outcomes in Scotland are collated centrally providing an extremely robust data set for investigation.

Furthermore, one quality indicator for intrapartum care, the 5-min Apgar <7 in term babies [6], was higher in Scotland than other settings [10]. Data from the Information Statistics Division (ISD) Scottish Morbidity Record (SMR 02) show a relatively high proportion of term births with 5-min Apgar scores <7 of 1.1% in 2012. The Apgar score is based on a standardised clinical assessment of the infant’s condition at birth, using a scoring system in 5 categories scoring 0–10 at 1 min, 5 min and 10 min post birth. The 5-min Apgar <7 rate is an important measure of intrapartum care as it is associated with a considerably higher rate of cerebral palsy in later life [21] and observational data suggest that it can be improved by training [10]. Moreover, the Low Apgar rate appears to be independent of maternal demographics [22], which makes direct comparison of Units useful.

### Null hypothesis

The PROMPT training has no effect on the rate of term infants born with an Apgar <7 at 5 min observed when implemented at scale across the Scottish maternity health service.

To test this hypothesis we have designed and implemented the THISTLE Study- Trial of Hands-on Interprofessional Simulation Training for Local Emergencies.

## Methods/design

### Study design

The study is a multi-centre stepped-wedge clustered randomised controlled superiority trial (SW-RCT) [23–25] .

This design has been chosen because there was not sufficient equipoise amongst participating centres about the intervention. So far, observational studies have consistently shown that PROMPT training is consistently associated with positive outcomes - indicating at least absence of clinical equipoise compared to no treatment and suggesting, albeit risk of confounding and selection biases associated with observational study design a potential treatment effectiveness. It was therefore deemed unethical to use a traditional parallel cluster RCT design in which some Maternity Units and their patients would have been allocated to no training. Furthermore, there was no obvious choice for an alternate training strategy to deliver in a control group as other similar training programmes have either been associated with harm [7–9] or have no evidence with regards to their effectiveness and safety. Several Units had also requested the training prior to this study and the PROMPT training was supported by a Scottish Government initiative. A SW-RCT was considered as the safest and most ethical option allowing us to roll out the intervention to all Units whilst still using randomisation, and with a number of practical and logistic advantages including modest costs and staffing requirements.

Finally, the established practice of routinely recording birth outcomes across all Scottish Maternity Units (ISD SMR 02) also allowed access to good quality data. The SMR02 is regularly updated reducing the burden on participating Units to provide outcomes specifically for this trial at each time-period and therefore the risk of incomplete data, facilitating the implementation of a SW-RCT.

**Fig. 1:**
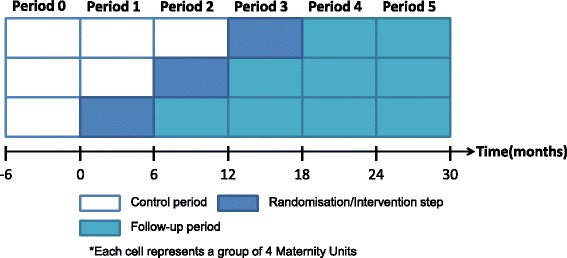
Stepped wedge-design of the THISTLE study

Details of the study design are presented in Fig. 1. The study has one control period (step 0) during which none of the Units receives training, three intervention (steps 1–3) and two follow-up (step 5) periods, each lasting 6 months. This duration allows all 12 Units to receive their training (see section on intervention) within 12 months, keeping each step short to reduce contamination between trained and untrained Units (rotation of trained staff between Units) but also to remain within the funding and time constraints while allowing for follow-up periods. Based on previous experience [16, 26] it was anticipated that Units would be able to train all of their staff within one year of commencing their local training. Four Maternity Units are randomised to each intervention step, i.e. the intervention is rolled-out sequentially to the Units in groups of four.

### Study duration

**Fig. 2:**
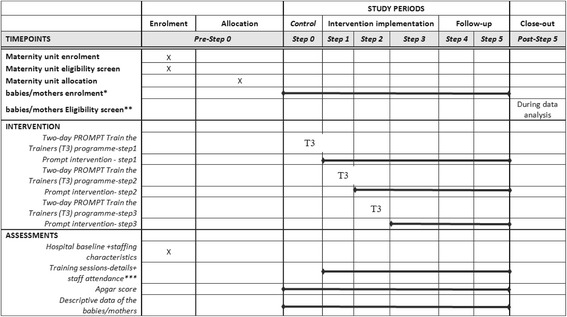
SPIRIT flow diagram for the Thistle study, a stepped-wedge clustered randomised controlled superiority trial

The study commenced in March 2014 (training of the first 4 participating Maternity Unit trainers), with step 1 starting on 1st of April 2014 and step 5 finishing the 30th of September 2016. It was unnecessary to begin the study with the control period factored in (Fig. 1) as the use of historical, routinely collected data from the ISD will allow to retrospectively access information on each birth from 2000 onwards. Further details of the study timeline are provided in Fig. 2.

### Participants

#### Settings

The study is being conducted across the NHS Scotland Maternity Units. Eligible Scottish Units were identified have been invited to participate through the Scottish Committee Royal College of Obstetricians and Gynaecologists.

#### Inclusion criteria

The study population consists of the 15 Scottish Maternity Units with >900 births per year. Participating Units that have not previously undertaken PROMPT training (*n* = 12) have been randomised and will be part of the main analysis.

The three Units that have already undertaken PROMPT training will not be randomised but will be included in complementary analyses. Their inclusion will allow us to assess the intervention effect at a national scale, whilst giving us insight into the long-term sustainability of the intervention effect. The primary outcome will be derived for each of the 15 Maternity Units.

#### Exclusion criteria

Scottish Maternity Units or Midwifery Units with <900 births per year are ineligible for recruitment.

### Intervention

#### Intervention to be evaluated

The intervention is the 2 day PROMPT Train the Trainers (T3) programme. It includes a demonstration PROMPT course, a Train-The-Trainers (T3) day and the local use of the PROMPT Course-in-a-box (Second Edition) [27, 28]. The Train-The-Trainers (T3) day includes lectures and workshops for electronic foetal monitoring and simulated emergency scenarios that instruct in-house trainers in how to set up training in their own Unit. The Implementation package has been available since January 2008 and provides tools and guidance to Maternity Units on how to implement annual, multi-professional, intrapartum emergencies training by ‘in-house’ trainers for all maternity workers in their own Units.

Each Maternity Unit participating in the THISTLE Study has been requested to identify four multi-professional in-house trainers (a team comprising 2 x Midwives, 1 x obstetrician and 1 x anaesthetist), who were then invited to attend the two-day T3 programme at The Scottish Clinical Simulation Centre in Larbert, before the start of their allocated step (March 2014 for Units allocated to step1, July 2014 for those allocated to step 2 and November 2014 for those allocated to step 3).

Each local multi-professional team is provided with a PROMPT Course-in-a-box (Second Edition) containing course materials and information for the implementation of training [27, 28]: Trainers Manual, Course Manual and a DVD which includes tutorials, clinical and team work checklists, clinical algorithms and demonstration videos. The local training teams will be provided with telephone/email assistance as well as practical advice and support regarding setting up their local training.

After participating in the 2-day T3 programme, in-house trainers are given time to set up and commence implementation of in-house PROMPT courses locally. They are requested to aim to have trained all maternity staff in their Unit within a year of commencing training, although this will be at the discretion of each individual Unit. The aim is to pragmatically assess implementation, thus determining the true effect and sustainability of PROMPT training.

The local in-house PROMPT course is a one-day, simple, adaptable multi-professional training course that covers the management of obstetric emergencies such as eclampsia, post-partum haemorrhage, maternal collapse, sepsis and shoulder dystocia. In-house training includes a variety of teaching methods including lectures, video demonstrations, and multi-professional rehearsals of simulated obstetric emergencies, using simple tools and algorithms to make it easier to implement the correct treatment. All staff should be trained annually.

#### Standard care

Standard care is expected during the control period for all Maternity Units (step 0) and also the intervention periods for Units not allocated to steps 1 and 2. All Units will be considered to have stopped standard care at the start of step 4 (last intervention period).

#### Follow-up

Maternity Units will all be monitored for at least 12 months from the end of their intervention period, until the end of the last follow-up period (step 5). All mothers and children will receive clinical follow-up as part of their standard care, but we do not intend to continue any follow-up beyond birth for the purposes of this study.

#### Safety

The intervention being evaluated is available to NHS and international maternity staff. With the assistance of the Royal College of Obstetricians and Gynaecologists (RCOG), the PROMPT ‘Course in a Box’ was first published in 2008 (PROMPT ‘Course in a Box’ 2nd Edition published in 2012), and has no known safety concerns.

### Outcome measure

#### Primary outcome measure

The primary outcome measure is the proportion of infants born with a low Apgar score (score < 7 vs. ≥7) at 5 min for all vaginal or emergency CS term livebirths (≥37 weeks). The Apgar score is routinely assigned for all births and collected at Unit level with later collation at national level. The primary outcome will not be derived for babies born at home or at other hospitals before being transferred to one of the participating Units, preterm infants and elective Caesarean Section (CS) births. Intra-uterine deaths diagnosed prior to labour will also be excluded from the analyses.

#### Secondary outcome measure

None

#### Process measures

Descriptive data from the Units will be collated:

- Total number of live births per period
- Total number of staff in the Unit – separated into staff groups
- Total number of staff trained – separated into staff groups
- Process measures: % of staff trained and time to train local staff

#### Data collection

Details of the data collection are provided in Table 1.

**Table 1:** Source and timing of collected data

Outcome variables	Source	Timing
Descriptive data of the Units	Individual Maternity Unit	On sign up to the THISTLE study
Total number of staff-separated into staff groups	In-house coordinator/Lead clinician	On sign up to the THISTLE study
Time period to train ALL local staff	In-house coordinator	Monthly after in-house trainers have attended T3 course
% of staff trained	In-house coordinator	Monthly after in-house trainers have attended T3 course
Apgar score at 5 min for each vaginal and emergency CS term birth	ISD SMR 02	Before the training (retrospective data back to year 2000) and then extracted regularly throughout the study
Descriptive/demographic data of the babies/mothers	ISD SMR 02	Before the training (retrospective data back to year 2000) and then extracted regularly throughout the study

These data will be collected for all 15 Units, except for the information relating to the implementation of the intervention, which will only be collected for participating Units who have not previously undertaken PROMPT

Each Maternity Unit will provide the study office with a list of dates for all of their local PROMPT Courses for the calendar year over which the training is anticipated to run. Units have been provided with a Thistle Study Follow-up pack containing information and data collection sheets (Thistle Study - Local Training Record). The Local Training Records are completed by the Unit’s training coordinator and returned to Thistle Study office, as soon as possible after each of their local PROMPT Courses. It records the date of the local course and the numbers, grade, and job roles of staff attending. No personal information from Unit staff will be collected and only aggregated totals by professional group as shown in the previous section will be collected at Unit level. Staff should also include a copy of the course programme when sending back the completed form. These data will be entered onto the Thistle database by the study data coordinator. Data collected during the course of the research will be kept strictly confidential and accessed only by members of the study team. The sponsors are responsible for ensuring that study data is archived appropriately. Essential data shall be retained for a period of at least 10 years following close of study. The study will comply with the Data Protection Act 1998 and regular checks and monitoring are in place to ensure compliance. Data are stored securely in accordance with the Act and archived to a secure data storage facility. The senior IT manager (in collaboration with the Chief Investigator) will manage access rights to the data set. Prospective new users must demonstrate compliance with legal, data protection and ethical guidelines before any data are released.

All demographic, clinical and outcome data will be extracted from the ISD SMR 02 [29]. The ISD of the National Health Service, Scotland, is a national organisation for health information, statistics, and computing services and has been in existence for over 40 years. Anonymised patient based data for maternity care are routinely collected from the whole of Scotland and this database (specifically SMR02) will allow us to identify eligible births. The database is subjected to regular quality assurance checks and has been more than 99% complete since the late 1970s. Information from the Scottish Birth Record, another data source maintained by the ISD recording every baby born in Scotland but less exhaustive than the SMR 02, will also be used to complete information relative to the Apgar score missing in the SMR 02 [30].Data will be accessed remotely using the National Services Scotland Safe Haven, a secured facility allowing authorised researchers to analyse individual-level data while maintaining the utmost confidentiality. Permission to use the data has been approved by ISD (XRB13180). These data will be saved on the Safe Haven, only accessed by approved member of the research team (EL) and will be archived as per ISD regulations.

Maternal/infant characteristics will be limited to the date of birth and the Apgar score at 5 min. Preliminary research conducted by the co-applicants on term births in one Bristol local hospital have revealed the limited interest of risk adjusting the analysis of Low Apgar status at 5 min for maternal socio-demographics characteristics. To select the relevant population of term infants (see exclusion criteria), the following factors will be considered: type of birth (vaginal, emergency or elective CS); duration of gestation (<37, ≥37 weeks); place of birth (at home, transfer from a non-NHS Unit, transfer from a NHS Unit, in the Unit of interest); and intra-uterine deaths. We will extract the information from 2000 to the end of step 5. There are no patient-reported or economic outcomes.

Perinatal outcome data from the ISD SMR 02 will continue to be collected from participating Units irrespective of whether the PROMPT T3 training programme is undertaken and local PROMPT courses are implemented within the time span of the study.

### Sample size

Of a total of fifteen Maternity Units with >900 births per year in Scotland (in 2010) three Units have already undertaken the PROMPT Train the Trainers day and had started implementing the intervention prior to the start of this study. These three Units are excluded in the following power calculation which focuses on the remaining eligible 12 Units that will contribute data to the before and after intervention periods. Annual birth numbers per Unit are available via an anonymised aggregated Maternity Unit dataset from SMDR for the period 2004–2010. We have made a conservative assumption that the Maternity Units which have already undergone PROMPT training are the three largest Units in the anonymised data. The 12 remaining Units had an Apgar < 7 rate at term of 1.18% in 2010 and an average monthly 200 births per Maternity Unit, i.e. around 1200 births per semester (duration of a step).

Using the method to calculate power for Stepped Wedge designs defined by Hussey and Hughes [25] and its Stata implementation [31], and considering that all available and eligible data for the considered Maternity Units will be analysed, the following intervention effectiveness can be detected: With a power of 80% (alpha = 5%), four Maternity Units randomised at each step with three intervention steps - steps 1 to 3 - (12 Units(clusters) in total) and two follow-up steps - steps 4 and 5 -, of six-month duration each (steps 1 to 5), an average cluster size of 1200 births, and an intra-cluster correlation coefficient of 0.1, we will be able to detect a reduction of 35% or more in the Apgar < 7 rate (i.e. 1.18% vs 0.77%).

### Randomisation

One Maternity Unit, independently undertook the PROMPT T3 training a few weeks before the start of the study but had not commenced in-house PROMPT courses and was therefore constrained to be allocated to Step 1 to allow inclusion of this Unit in the study.

The remaining participating Units are allocated to the intervention at one of the “randomisation steps” (step 1, 2 or 3 see Fig. 1) using the “imbalance statistic” method [32, 33]. This approach permits the balancing of the steps by number of births per annum (small, medium, large Units). An independent statistician from the Centre for Healthcare Randomised Trials (CHaRT) will randomly select an allocation sequence from a subset with the most desirable balance properties using computer-generated random numbers. CHaRT will then inform the nominated in-house training coordinator from each Unit of the date that their team had been allocated to attend the T3 programme.

If the allocated step does not suit the local circumstances of a Maternity Unit (e.g. inability to release clinicians at the allocated training dates due to a shortage of staff), the step allocation is reconsidered. As mentioned by Handley et al. a more pragmatic approach is possible in a stepped-wedge design, by for example considering a manual assignment of Units to steps [34]. To continue to ensure an equal balance in the size of the Maternity Units in each step, if a Unit is unable to attend the allocated training, this Unit will be assigned to a more convenient step, but swapped with another of a similar size wherever possible.

Pregnant women will not be made aware of Maternity Unit status regarding participation in the study, and any additional training that each Unit would normally undertake, should continue.

### Blinding

Due to the nature of the intervention and the choice of study design, the staff and research teams could not be blinded to the Unit’s training status.

### Statistical analysis

#### Pre-intervention period investigation

The secular background trend before the initiation of the study of the rate of term Low Apgar at 5 min will be described from the ISD SMR data using births between 2000 and the beginning of step 0.

#### Participation, loss to follow-up and withdrawal

Analysis and presentation of data will be in accordance with CONSORT guidelines and recommendations for Stepped Wedge available at the time of reporting [24]. Unit recruitment, in-house trainers’ participation to T3 training, and in-house PROMPT course implementation will be documented. Loss to follow-up will only occur in the unlikely event of a Maternity Unit closure.

Units that withdraw, or do not comply with the intervention, will be analysed on an intention-to-treat basis. If a Unit does not attend their allocated train the trainer session during the THISTLE study intervention or follow-up period, that Unit data will be excluded from the primary analysis (See section on sensitivity analysis). We do not anticipate extensive missing data for the primary outcome and our analysis strategy will be on complete cases. However, it is known that for a very small number of births, the Apgar score is not collected. We will describe any missing data in detail, and if required, will test the robustness of our primary analysis using appropriate imputation strategies [35].

#### Baseline characteristics and intervention delivery (process measures)

Appropriate descriptive statistics will be used to summarise all baseline characteristics. The number of staff (total and by professional group), number of PROMPT courses delivered by in-house trainers and the length of time required to train all the staff will also be reported for each of the 12 Units.

#### Main analysis

The number of births (count) and primary outcome will be tabulated by step (0 to 5) and by Unit. The primary outcome will be plotted for each Unit as well as globally for all Units to explore patterns visually. The primary outcome will be modelled at the level of the individual birth and analysed with a mixed (Generalized linear Mixed models strategies) or marginal (Generalized Estimating Equations) logistic regression model [36, 37] depending on encountered convergence or computational issues. This will allow us to account for the correlations between births occurring in the same Maternity Units. Initial models will assess the intervention effect (control period/step vs. intervention effect/step considering follow-up steps as intervention steps) and be adjusted for the effect of time-period to account for potential confounding effect of time, i.e. an underlying time-trend in the prevalence rate of Apgar score < 7 during the study period [38]. We will model the time-period using dummy indicators for each step. The interaction between time and the main intervention effect will then be tested to investigate the timing and duration of the intervention effect. All clusters will be analysed as randomised, i.e. we will make no allowance in these analyses for any Units that did not implement the intervention as per randomisation schedule. No interim analysis is planned, one final set of analyses will be conducted after the end of step 5, and as soon as the data are available.

#### Sensitivity analyses

Sensitivity analyses include:

- Per-protocol analyses, to reflect Units that do not (fully) comply with the intervention at their allocated step. We will replicate the above analyses but use the actual date of implementation of the first training session to define the control and intervention periods of each Maternity Unit. In these analyses, only the Maternity Units that have implemented at least one training session will be considered.
- Adjusted analysis to test the robustness of any potential imbalances (low Apgar scores in the pre-study period; Maternity Unit volume).
- Investigation of the time-trend effects and timing of the intervention effect.by modelling steps of different lengths.

#### Additional analyses

To provide an assessment of the intervention effect across the health service, the 15 main Scottish Maternity Units will be considered in the next set of analyses. This will include the three Units trained prior to the start of the THISTLE study and the 12 Units targeted by this study, including those that have not implemented any local PROMPT courses.

Data from the three Units previously trained will be considered as part of the intervention periods throughout the analyses. Those Units not implementing any training will be considered as part of the control periods throughout the analyses. The data of the other Maternity Units will be considered as either part of the control or interventions periods depending on the date of implementation of their first local PROMPT course.

Finally, to provide an exhaustive understanding of those results, the 6-monthly rates of Low Apgar scores, scaled on a similar timing as the steps defined in Fig. 1, will be derived. They will be plotted for each of the 15 Units. These graphics will help to:

1. understand any interactions between time and intervention effect as discussed above
2. explore the impact of the heterogeneity in the intervention implementation (timing and frequency of training sessions).

We will use an a priori assumption that the background rates of 5-min Apgar <7 among the 3 Maternity Units already trained will be lower than the other Units; and the Units which have not implemented any training are expected to have higher rates.

This graphic and two sets of sensitivity analyses will be used to discuss the relevance of the main analysis (and its extension) and determine the external validity of its findings, i.e. to what extent the findings can be generalised across the health service.

Studied centres have been followed until the 30th of September 2016 and related data will be accessible in March 2017.

## Discussion

This paper describes a stepped-wedge clustered randomised controlled trial designed to evaluate the effectiveness of a multi-professional intrapartum emergencies training course. The aim is to establish whether the PROMPT training can improve perinatal outcomes.

Findings from previous observational studies have shown promising effects associated with PROMPT training, but the impact of residual confounding and degree of external validity associated with its implementation in enthusiastic, early adopter single-centres are unknown and prevent the accurate assessment of the true effectiveness at scale. The stepped-wedge clustered design allows the investigation of one of the few obstetric interventions with observational evidence from multiple sources of local success in different settings, in respect with the issue around equipoise. This also allows a pragmatic investigation of the intervention in the whole Scotland, efficiently capitalising on existing routinely Maternity datasets for which no individual consent is required. Our robust methodology should provide useful information for both clinicians and policy makers.

### Trial status

The THISTLE Study received permission to conduct research on 01/08/2013. The “theoretical” date of first enrolment corresponds to the start of the control period, i.e. 01/10/2013 with the recruitment completed on the 30/09/2016. The final data extract is accessible since 20/03/2017 and analyses are currently ongoing.

## Abbreviations

Apgar: Medical assessment of newborn: - Appearance, Pulse, Grimace, Activity, Respiration
CHaRT: Centre for Healthcare Randomised Trials
CONSORT: Consolidated Standards of Reporting Trials
CS: Caesarean Section
ISD SMR 02: Information Statistics Division – Scottish Morbidity Records- Standard dataset for Scottish Maternity Units
ISD: Information Statistics Division
ISRCTN: International Standard Randomised Controlled Trial Number
NHS: National Health Service
PROMPT: PRactical Obstetric Multi-Professional Training
RCT: Randomised Controlled Trial
SW-RCT: Stepped-Wedge Randomised Controlled Trial
T3: PROMPT “Train-the-Trainers” programme
UK: United Kingdom
UKCRC: United Kingdom Clinical Research Collaboration
